# Healthy aging through the lens of community-based practitioners: a focus group study

**DOI:** 10.1186/s12877-020-01611-x

**Published:** 2020-06-15

**Authors:** Rubee Dev, Oleg Zaslavsky, Barbara Cochrane, Thomas Eagen, Nancy F. Woods

**Affiliations:** 1 Sun Yat-sen University Global Health Institute, School of Public Health, Sun Yat-sen University, Xingang West Road, Guangzhou, 510080 China; 2 Biobehavioral Nursing and Health Informatics, University of Washington, Seattle, WA USA; 3 Child, Family, and Population Health Nursing, University of Washington, Seattle, WA USA; 4 Rehabilitation Science and Health Systems & Policy, University of Washington, Seattle, WA USA

**Keywords:** Healthy aging, Community-based practitioner, Perspective, Focus group discussion

## Abstract

**Background:**

Nearly one in every seven Americans is 65 years and older, facing day-to-day challenge of aging. Although interest in healthy aging is growing, most of the efforts are directed towards understanding the perceptions of older adults. Little is known about the perspectives of community-based practitioners who work with older adults and deliver programs to promote healthy aging. The purpose of this project was to expand knowledge on healthy aging by exploring the perspectives of community-based practitioners working directly with older adults.

**Methods:**

We purposively sampled community-based practitioners (*n* = 12, including nurses, physician, social workers, and other community services professionals) working with older adults, who then participated in one of three in-depth focus group discussions conducted between March and June 2016. Each focus group discussion lasted for about 2 h. Verbatim transcript data were analyzed in Atlas.ti 7 using a conventional content analysis with an inductive approach, and consensual validation of coding was achieved.

**Results:**

Three core categories of healthy aging were identified: (1) characteristics of healthy aging; (2) healthy aging attainment; and (3) programs and activities for healthy aging. Practitioners identified a number of characteristics of healthy aging under person-specific (physiological, basic, psych-emotional, and cognitive needs), social aspects (creating community and contributing to the community), and spiritual dimensions (cultural views and beliefs) of healthy aging. Healthy aging attainment was represented as facilitators and barriers both with respect to care recipients and care providers, and programs and activities through promoting fitness and wellness.

**Conclusions:**

The rapidly changing demographics and aging population in the United States and their various needs suggest the implications for recognizing opportunities and developing and implementing programs to promote healthy aging. Although practitioners’ perspectives had some overlap with traditional research and medical views on healthy aging, the unique and holistic conceptual framework derived in the study might provide a more refined foundation for delivering appropriate health care services to the American aging population.

## Background

Nearly one in every seven Americans is 65 years or older and faces the day-to-day challenges of aging [1]. The growing proportion of older adults in the U.S. provides a compelling reason for an increased focus on aging well and strategies to optimize the experience of aging. Demographic patterns in the U.S. have become dramatically more diverse, prompting a consideration of the ethnic and economic changes in health disparities of aging services and supports [2]. This diversity of views regarding contexts for promoting healthy aging merits an examination at the intersection of various aspects of older adults’ lives such as race, socioeconomic status, living situation, physical and emotional health, spirituality, and other dimensions. A range of approaches for defining healthy aging from the perspectives of older adults and persons studying aging have been evaluated in the literature [3–6]. These perspectives are rooted in distinct traditions of theory and empirical work that inform current practices to support aging [7]; however, little is known about the perspectives of community-based practitioners’ who work directly with older adults and deliver programs to promote their healthy aging.

The term healthy aging is widely used in academic and research circles, yet there is limited consensus on how it might be defined [8]. Among related theoretical constructs studied historically, “successful aging” was defined by Rowe and Kahn as freedom from disease or disease-related disability, high cognitive and physical functioning, and active engagement with life [9]. Similarly, “effective aging” was suggested by Curb et al., 1990 as an alternative to successful aging in order to emphasize the adaptation and rehabilitation that can occur even as older adults develop health deficits (e.g., chronic conditions, disabilities) [10]. Finally, “optimal aging” was exemplified by Ryff’s work focusing on psychological thriving and well-being [10–12]. Although these perspectives complement the construct of healthy aging, they vary widely in its measurement as an outcome. In 2015, the World Health Organization defined healthy aging as the process of developing and maintaining the functional ability that enables well-being in older age [13]. While these perspectives of aging have informed clinical, research, and policy implications, they are still underutilized in practice, potentially because of low “buy-in” from stakeholders such as those who implement health promotion programs for older adults.

The appropriate alignment of community-based practitioners’ perspectives to the needs of older adults plays a vital role in supporting healthy aging. However, most research efforts have been directed towards understanding the perceptions of older adults and scholars of aging [14–17], not the community-based practitioners delivering services and supports directly to older adults. As a result, our current knowledge remains limited from the community-based practitioners point of view. Older adults’ perspective of healthy aging emphasizes factors that practitioners may or may not necessarily consider during service provision.

Considering the existing health problems of older adults and lack of evidence-based research related to the practitioners’ view on healthy aging, we conducted a qualitative perspective assessment of community-based practitioners. Practitioners’ perspectives on healthy aging and strategies they employ to promote aging can enhance our understanding of the complexities associated with care at the community level and inform interventions to maximize older adults’ healthy aging experiences. Such understanding may also contribute to building a knowledge base of possible interventions for future healthy aging policies and programs. Hence, the purpose of this study was to fill an important gap in the healthy aging literature by expanding knowledge on healthy aging by exploring the perspectives of community-based practitioners working directly with older adults.

## Methods

### Participants

Participants were recruited through the de Tornyay Center for Healthy Aging at the School of Nursing, University of Washington. The center serves as a catalyst for promoting healthy aging through advance nursing science and practice by providing support to faculties in conducting research related to healthy aging and in building community partnership [18]. Using purposive sampling, diverse community-based practitioners specifically engaged in providing a variety of services and programs for older adults in King County, Washington, were invited by email to participate in focus group discussions (FGDs). Purposive sampling was used as it allows researchers to decide what needs to be known and find participants who can and are willing to provide information by virtue of knowledge or experience [19]. If interested in the study, potential participants were screened by phone for the following eligibility criteria: age 21 years or older; worked at least part-time (4 or more hours/week) directly with older adults or with organizations, facilities, or activities that served older adults; and able to speak/understand English. A total of 16 potential participants working in senior living and community service agencies, including home health care and local area agencies on aging were contacted. Of these, 12 participants met inclusion criteria and were interested in participating, and all 12 were scheduled to attend focus group discussions. Participants represented a range of community-based practitioners, including nurses, social workers, and other community-based professionals who directed or delivered programs and services to older adults. Two researchers (BC and OZ) trained and experienced in qualitative methods and interested in aging research conducted the focus groups. Demographic characteristics of study participants are presented in Table 1.

**Table 1:** Participant demographics (*N* = 12)

Characteristics	% of participants (n)
Age (years)	
≤ 55	25 (3)
> 55	75 (9)
Gender	
Female	100 (12)
Race/Ethnicity	
White (not Hispanic)	92 (11)
Black (African American)	8 (1)
Disciplines	
Medicine	8 (1)
Nursing	50 (6)
Social work	15 (2)
Gerontology/Other	25 (3)
Current community-based practice focus	
Community services agency	58 (7)
Primary provider	15 (2)
Senior living	25 (3)
Education	
Baccalaureate	42 (5)
Master	25 (3)
Doctoral	33 (4)

### Procedures

Data were collected between March and June 2016 with the sample of 12 community-based practitioners who participated in one of three focus group discussions, each approximately 2 h in length, at a conference room at the University of Washington School of Nursing. Each focus group involved four participants. The discussions were led by a facilitator; a note taker from the research team was also present. The focus group facilitator informed the participants about all study procedures and obtained written informed consent. Participants were then asked to fill out a brief questionnaire about their background (e.g., age, gender, family income). The facilitator reminded participants about focus group considerations (e.g., confidentiality, audio-recording) and then began the discussion with the general question, “What does “healthy aging” mean to you?” before asking other semi-structured questions (Supplementary file 1), as well as questions that sought to clarify and elaborate evolving themes. Based upon the research objectives and researchers’ discussion, list of questions was prepared as a guidance for each focus group discussion session. Participants received a $25 gift card for participating. Each discussion was recorded digitally and transcribed verbatim. All audio-recordings were destroyed after transcripts were verified. The transcript data were then uploaded into the Atlas.ti 7 software program (Cleverbridge, Chicago, IL) for data management and analysis. Atlas.ti was used as it is among the best available and potentially most useful qualitative data analysis tools [20].

### Data analysis

A conventional content analysis with an inductive approach was used to analyze the verbatim transcript data. This approach is appropriate when existing theory or research literature on a phenomenon is limited, allowing researchers to immerse themselves in the data to allow new insights to emerge. The process is also described as inductive category development [21]. Decisions about further sampling, data collection, and coding were guided by a goal of achieving data and thematic saturation, when no new data or themes emerged from the discussions. Saturation in this study was considered as a point at which no new codes occurred in the data and additional data did not lead to any new emergent themes [22].

**Fig. 1:**
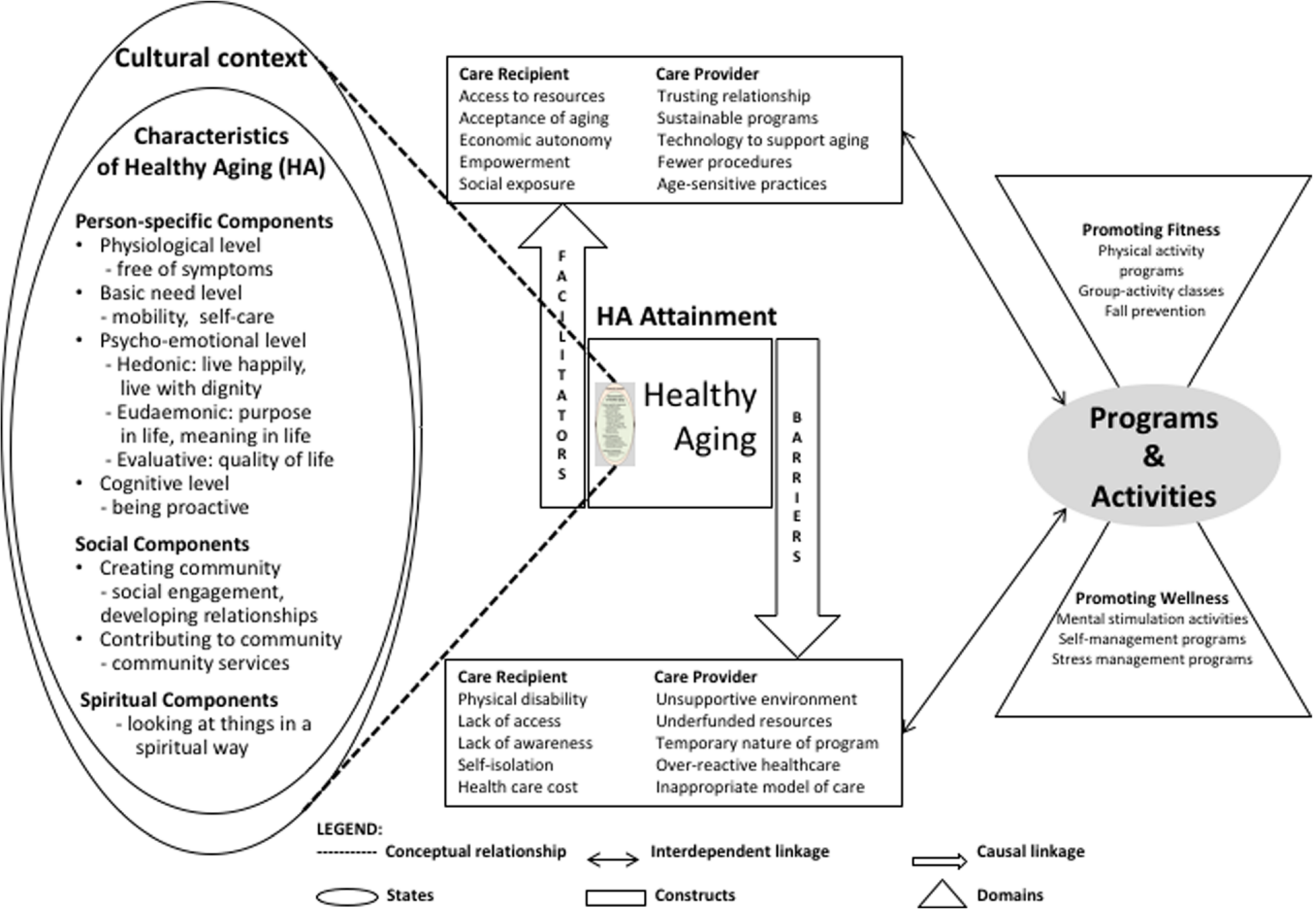
Conceptual framework of the perspectives of community-based practitioners’ on healthy aging

Analysis occurred in four phases following the guided analytic cycles of qualitative data analysis [23]. In the first phase, the first and second authors (RD and OZ) quantitatively inspected the data and independently coded the transcripts using open coding and then met to discuss their individual codes, identify areas of disagreement, and reach consensus after discussing meanings to establish inter-coder agreement. In the second phase, the coded experiences and opinions of all the practitioners were grouped and categories were drawn out to derive a comprehensive perspective on healthy aging. In the third phase, all researchers employed a member-checking strategy that involved summarizing information to attendees (including original focus group participants) at a gerontological professionals network meeting, obtaining their input, and thus affirming the accuracy and completeness of our findings to enhance the credibility of the findings and validate responses. In the final analysis phase, a conceptual framework (Fig. 1) of healthy aging was developed synthesizing and corroborating findings that emerged from the data analysis.

### Trustworthiness

Use of Atlas.ti software contributed towards enhancing rigor in this study by supporting rigorous open coding process, classifying and re-arranging emerged codes through a feature called network building, and exploring the complex phenomena hidden in the gathered data through analyzing the conceptual relationships [24].

## Results

The mean age of the participants was 55 years (range of 32 to 70 years old), and 92% identified their race/ethnicity as white (not Hispanic origin). Half of the participants were nurses and slightly more than half (58%) were involved in community services agency. All participants were female. A total of 42% had a higher than baccalaureate degree of education (Table 1).

Three core categories were identified from the transcripts: (1) characteristics of healthy aging; (2) healthy aging attainment; and (3) programs and activities for healthy aging. Figure 1 includes these core categories and their themes, depicting the conceptual dimensions of healthy aging and their relationships as identified from the perspective of community practitioners. Characteristics of healthy aging included three distinct themes: person-specific components, social components, and spiritual components. Healthy aging attainment was characterized by two distinct themes: facilitators and barriers. Two distinct themes related to programs and activities were promoting fitness and promoting wellness. Each of the core categories and associated themes is discussed in detail below.

### Meaning of healthy aging

Community-based practitioners defined healthy aging in terms of meeting the basic needs of older individuals (e.g., nutritional, housing, medical) and making sure they have access to resources. Focus group participants emphasized, “*resources are a broader term than just financial resources that includes personal resources, physical wellness to be able to live independently and be socially connected.*” Additionally, participants indicated that the meaning of healthy aging would differ depending on the individual characteristics of an older adult. One participant commented:

*“So, for people with reasonable funds … I would think healthy aging is more staying active, making sure that you’re doing what you want to do that brings value to you to the extent that you want to do that, you live where you want to.”*

Some participants reflected on healthy aging as older adults being able to do things that are meaningful to them and maintain meaningful relationships within their community, family, and religious groups. They further added exploring new experiences, discovering new things, and finding new avenues of expression and joy.

### Characteristics of healthy aging

The various characteristics of healthy aging derived included: (1) person-specific components at the individual level, (2) social components that encompass interactions with one’s community, and (3) spiritual components that incorporated more transcendent perspectives on growing older.

#### Person-specific components

Participants discussed various person-specific components including: (1) physiological – being free of disease-specific symptoms; (2) basic needs – being able to manage self-care independently and free of disabilities that hamper mobility; (3) psycho-emotional – subjective well-being through hedonic, eudaemonic, and evaluative approaches; and (4) cognitive – being proactive and having conscious control over one’s life.

*“I mean … at the early stage, people don't necessarily need a caregiver. It's about that, being able to be proactive and about having the positive attitude is critical in maintaining quality of life for that period of one to five years.”*

#### Social components

Most focus group participants identified social components as key to healthy aging, with available and accessible opportunities for socialization. The social components were classified by the older adults’ type of involvement, either creating or contributing to their community (e.g., engaging in community services).

*“I would agree with the basic needs, and I’d add the social component, which is key, I think, to healthy aging … I mean it’s one thing to live independently, but if you’re not connected to others, whether it be for fun or for support with daily activities of living, I just think that’s a key component.”*

#### Spiritual components

Participants believed the spiritual component to be another key factor that determines whether a person is aging healthily. They viewed older people as having more time than younger adults and able to look at things with a more spiritual and positive perspective.

*“Healthy aging to me is exploring new experiences and finding new avenues of expression and joy. There’s a lot of people that are now trying to change the negative image of aging and focus on more positive aspects of aging. I think that the spiritual piece is a part of that.”*

### Healthy aging attainment

The likelihood of healthy aging attainment depends on various factors. These practitioners identified those factors in terms of facilitators and barriers towards healthy aging, which were related to care recipients and care providers. The following are some of the facilitators and barriers that were commonly associated with the attainment of healthy aging:

#### Facilitators of healthy aging

##### Care recipient

Equipping older adults with resources was identified as a facilitator that gives them hope to potentially meet their goals. As one participant said, “*There are resources for adaptation that will make the elderly live more successfully and keep them independent for a longer period of time.”* Acceptance of aging was another key facilitator for older adults to make lifestyle decisions that take into account accepting their physical and social situation. Further, increasing their access to economic autonomy, enhancing social exposure, and making them empowered to make their own choices in life were identified as facilitators of healthy aging by the practitioners.

##### Care provider

Developing a trusting relationship with health care providers and having program sustainability were considered important facilitators. Participants indicated that many opportunities to support aging lie in technology as well as the built environment. These professionals also advocated for fewer procedures at health care encounters, more age-sensitive health practices across settings, and sustainability.

*“I mean I’ve heard of some opportunities where some grants, like breast cancer awareness and stuff, and churches have been able to take advantage and apply for grants and receive funding, but then it comes, and it goes, and that's it. So, if there was something that could be … more stable, sustainable, and that's getting people where they are … . I think there's a lot of room for growth … sustainable funding is the key.”*

#### Barriers to healthy aging

##### Care recipient

The presence of disability, “aging-unfriendly” communities, and lack of access and awareness of resources makes it difficult for older adults to benefit from available services. In addition, increasing health care costs and the cost of supportive housing aggravate the restraints to achieving healthy aging.

*“More and more hospitals are being seen as cost centers, and your relative or you don’t want anybody to be in the ED or in the hospital because that just costs so much more than any other care … ”*

Furthermore, social isolation was identified as an emerging issue and more highly prevalent among older adults. Many elders were described as having a reduced social network and interactions, leading to a higher level of isolation, immobility, and lower well-being.

*“You'd see these giant apartment buildings just full of people and you think, oh, they're surrounded by people. They're surrounded by people, but they're completely alone and they can't get out for reasons we've been talking about … And there's nowhere for them to walk to if they did.”*

##### Care provider

Underfunded resources and the temporary nature of some programs due to lack of sustainability were viewed as major issues that are affecting the health care system and programs that support older populations. Healthcare services provided to older populations were also identified to be reactive healthcare – reacting to adverse conditions or symptoms and mainly treatment-focused as opposed to patient-centered, a practice described as deeply embedded in the healthcare system.

*“We’ve medicalized dementia. And the behaviors are upsetting for people. If they have to go to hospital, then they start vocalizing, and the hospital puts them on Risperdal – a medication that has a black box warning. It takes time to discover why they’re vocalizing.”*

### Programs and activities for healthy aging

Participants indicated that as people age, some of them start experiencing limitations in activities and cognition. Senior centers were identified as providing a wide variety of services that help older adults meet their daily living needs. Two themes emerged related to programs and activities used to promote healthy aging: (1) promoting fitness, and (2) promoting wellness.

#### Promoting fitness

Participants discussed programs that promote fitness through services involving meaningful physical activity -- not necessarily “exercise” but whatever keeps older individuals engaged. These professionals emphasized, in particular, the importance of group activity classes and fall prevention programs.

#### Promoting wellness

Participants also discussed mental stimulation activities, self-management programs, and stress management programs to promote wellness, emphasizing mental wellness as a necessary component.

*“I think the mental health side really complicates things because so many people live with depression or anxiety … tendencies that they have lifelong and compensated well for. And then when they lose that ability to compensate, things can really spiral quickly.”*

## Discussion

In this qualitative study, we provide an in-depth understanding of healthy aging from the perspectives of community-based practitioners serving older adults rather than older adults themselves, which are underrepresented in approaches that explore this construct. To date, research on healthy aging has been framed by theoretical traditions, older adult perspectives, and/or clinical perspectives. While the earlier research has value in understanding older individuals’ health and well-being, it does not fully address the intersectionality of clinical, community, and individual characteristics, which can be obtained from those providing care and programs in the community and would be important for informing interventions to enhance healthy aging.

The conceptual framework derived from the focus group data in this study depicts the intersectionality between (i) individual-level characteristics, (ii) system-level facilitators and barriers, and (iii) community-level programs and activities to promote healthy aging. Characteristics of older adults that are dependent on a cultural context they live in lies as a basic component of healthy aging. It will be important to understand the contextual factor of each individual that will help in the delivery of culturally appropriate healthcare services. Further, it shows that healthy aging attainment could be enhanced by facilitators or hindered by barriers by a broad range of constructs such as access to resources and inappropriate model of care. Practitioners who could be aware of these constructs could act on the facilitators and barriers of implementing and delivering programs and activities. Finally, the framework highlights the evidence-based programs and activities that could promote fitness and wellness of older adults. This might be useful for the community-based practitioners in delivering evidence-based services to promote healthy aging, within and beyond the Washington state.

The framework incorporates many constructs of healthy aging that is in agreement or disagreement with the perspectives of older adults in the research literature. A study conducted among Chinese older adults in Chicago’s Chinatown to understand the culturally specific views of health reported individual characteristics that influence their perceived needs of health as physical function, psychological well-being, social support, and cognitive function. These constructs are similar to the constructs reported by practitioners in our study. Similar to the findings of our study, lack of access to resources and affordability of services were reported as major negative enabling factors that inhibited their health aging attainment. In addition, Chinese older adults also reported linguistic barrier as a major obstacle [25]; however, the linguistic barrier was not perceived as a serious hurdle by the practitioners in our study.

Further, the psycho-emotional dimension of healthy aging discussed by the practitioners, as exemplified by purpose in life, aligns well with the notion of optimal aging and eudaemonic well-being [11]. In addition, references to happiness and living with dignity reflect the hedonic notion of well-being as emphasized in work on happiness [26]. Older adults typically have developed improved emotion regulation as they age and have contemplated meaningful goals, reflected in hedonic and eudaemonic dimensions of well-being [26]. Cognitive function, exemplified by ability to be proactive and planning, is consistent with elements in each of these notions of successful, effective, and optimal aging. In addition, autonomy, competence, and relatedness have been identified by older adults as essential dimensions of self-determination for which cognitive function is essential [27]. Moreover, cognitive functioning is necessary for developing adaptation strategies and social interaction supporting engagement with one’s environment [28, 29].

Moreover, our analysis shows that with aging, many individuals incur losses from their social networks and experience stressors related to the broader context of their lives and changes associated with their own physical aging, and some experience neurological dysregulation [26]. These situations prompt a need to address the social aspects of healthy aging, such as social engagement and social support, similar to promoting social culture as pointed out by Lorek et al. [30]. In addition, the eudaemonic dimension of well-being gives rise to a need to create and contribute to a community for many, prompting engagement in volunteerism. Social components of healthy aging, including engaging with others in social events and contributing to the community, are consistent with notions of healthy aging as in Benson et al.’s proposed centrality of human relationships revealed through social support and social engagement [6].

Our findings also suggest that the spiritual component of healthy aging is commanding increased attention, with an appreciation of the importance of faith communities and individual spirituality, and their association with health [31]. Indeed, the capacity to transcend some aspects of aging, including losses, may reflect spiritual dimensions of health. The spiritual dimension of healthy aging was referenced by the practitioners as they reflected on more transcendent perspectives of older adults and the positive aspects of life.

In addition to these aging services practitioners’ perspectives being reflected in the research literature to date, there is also a close alignment between their views and those of older adult participants in studies of healthy aging. For example, Phelan and colleagues found that over 90% of older Japanese-Americans and Whites participating in longitudinal studies believe the following dimensions of successful aging were most important: remaining in good health until close to death, being able to take care of oneself until close to the time of death, and remaining free of chronic disease [32, 33]. Taken together, these perspectives from older adults bear close resemblance to those cited by the community-based practitioners who participated in this study.

There are a wide variety of programs and services available to promote healthy aging and independence among community-dwelling older adults (e.g., physical activity, fall prevention, self-management), which align with promoting fitness from our healthy aging framework. Nationally, beneficial and protective effects of physical activity are recognized across the lifespan [34]. Physical activity programs designed specifically for older adults have been disseminated and implemented widely through community partnerships [35]. The new multi-disciplinary approach to fall prevention is one example of how the healthcare system has modified processes to increase adoption of evidence-based programs that support healthy aging. The Centers for Disease Control and Prevention has provided physicians and nurse practitioners with a fall-risk screening and assessment toolkit to evaluate common modifiable risk factors associated with falls (e.g., gait and balance impairment, medication management, environmental hazards etc.) [36]. Community programs have also been developed to promote wellness among older adults through self-management skills. Self-management skills are critical to maintaining quality of life and independence. The Chronic Disease Self-Management Program is an example of a program designed to improve self-efficacy and independence among community-dwelling older adults [37, 38]. Changes in the healthcare system have sought to overcome barriers to support clinicians’ recommendations of appropriate programs, based on the needs of the individual. Practitioners can work with rehabilitation professionals (e.g., physical and/or occupational therapists) and community health workers to assist with the implementation of the recommendations [39].

As communities continue to elaborate healthy aging programs and services, there is a need to fully encompass the multitude of views on healthy aging held by people carrying out these programs and to adopt sensitive, valid, and reliable outcome measures that capture the broad construct of healthy aging. The proposed novel conceptual framework can guide community-based practitioners in the assessment of healthy aging as well as in the development and implementation of community-based interventions to promote healthy aging, and quality improvement and program evaluation of services and supports for older adults.

This qualitative exploration of community-based practitioners’ perspectives on healthy aging has strengthened and expanded the healthy aging literature beyond theoretical frameworks and input from older adults themselves. However, the study is not without limitations that should be considered when interpreting results. First, the data used in this study are from a small sample of practitioners that did not include the full range of professionals working with older adults in the community that may limit the generalizability of study findings. It is possible that a larger, more diverse sample could have produced different themes of healthy aging. However, the current study participants brought diverse perspectives in terms of educational backgrounds, length of experience, work settings, and interactions with older adults, and data saturation was confirmed by member-checks of the themes that evolved. In fact, participants compared and contrasted their perspectives and experiences with those of other colleagues and collaborators, as well as their own personal experiences of aging and caring for older parents or grandparents. Second, the sample was purposively selected from one large metropolitan area in the Washington state of US, and hence our results may not be generalizable to other practitioners working with older adults in rural areas or facilities in other states, as their views may be subject to varying degrees of social and economic influence. As our findings are situated in a particular context, it is not our intention to generalize to other groups and contexts. However, findings of this study may provide meaningful information to the gerontological researchers in other US states or other countries on which to base subsequent future studies. Furthermore, although the participants represented diverse disciplines and backgrounds in this study, which is an important consideration for ensuring that evolving themes are informed by relevant as well as divergent views, participants’ racial/ethnic diversity was limited in the current study. It is possible there are differences in how healthy aging is viewed by practitioners from broader racial/ethnic or cultural groups, particularly for diverse populations of older adults. More research is needed to explore these potential differences. Third, our study sample included all female practitioners, the perspectives of whom may vary from the male practitioners. Some health care professions (so-called support occupations) are female-dominated profession in the US such as nursing and social work [40], which could have led to participation of all female practitioners in the study. Fourth, there was a wide variation in the academic training level of study participants ranging from undergraduate to doctorate level. This could have led to the wide variation in the perspectives; however, it is noteworthy that diverse views of participants added depth and breadth to the study findings. Future studies are needed to explore the perspectives of practitioners stratified by demographic characteristics, particularly academic training level. Nonetheless, this qualitative study generated useful insights about the healthy aging from the perspectives of community-based practitioners that lay the foundation for future research on the well-being of older adults.

## Conclusions

In conclusion, this study demonstrated that community-based practitioners working with older adults bring a breadth and depth of perspectives on the construct of healthy aging that complement and expand existing theoretical and research traditions. These perspectives offer important considerations regarding the development and delivery of services and supports for older adults. The practitioners had varied perspectives on healthy aging and utilized multiple strategies to address functional declines in aging; however, they also faced similar challenges, mainly related to the sustainability of healthy aging programs. Given the changing demographic pattern of the aging population, practitioners are in a distinct position to promote healthy aging, such as advocating for their clients and recognizing opportunities for developing and implementing relevant, proactive programs. Future research should seek to study more ethnically diverse perspectives, from a broader base of community-based practitioners, which could lead to improved understanding of healthy aging and appropriate services for older adults.

## Supplementary information

**Additional file 1: Supplementary file 1.** Sample focus group discussion questions.

## Data Availability

The datasets used and/or analyzed during the current study are available from the corresponding author on reasonable request.

## Abbreviations

ED: Emergency Department
FGD: Focus Group Discussion
US: United States
