# Supplementary material for: Healthy aging through the lens of community-based practitioners: a focus group study
Source: BMC Geriatr. 2020 Jun 15;20:211. doi: 10.1186/s12877-020-01611-x (PMC7296747; doi:10.1186/s12877-020-01611-x)
Supplement: Supplementary file 1 — Additional file 1: Supplementary file 1. Sample focus group discussion questions. [file 12877_2020_1611_MOESM1_ESM.docx]

**Supplementary File 1**

Sample focus group discussion questions

1. What does “healthy aging” mean to you? What is your take on the concept of healthy aging?
2. How would you know or recognize healthy aging in someone in your community? What makes someone a healthy older adult?
3. How does a person attain healthy aging?
4. Are there particular activities or programs that you know about or have used in your work to promote healthy aging?
5. Are there activities or programs that you use for yourself for the purpose of attaining healthy aging?
6. Are there activities or programs that you know about for promoting healthy aging that you would not recommend for others? Yourself?
7. What are facilitators and barriers for promoting healthy aging in your community?
